# A new shielding calculation method for X-ray computed tomography regarding scattered radiation

**DOI:** 10.1007/s12194-016-0387-9

**Published:** 2016-12-26

**Authors:** Hiroshi Watanabe, Kimiya Noto, Tomokazu Shohji, Yasuyoshi Ogawa, Toshioh Fujibuchi, Ichiro Yamaguchi, Hitoshi Hiraki, Tetsuo Kida, Kazutoshi Sasanuma, Yasushi Katsunuma, Takurou Nakano, Genki Horitsugi, Makoto Hosono

**Affiliations:** 1 Department of Radiological Technology, Japan Organization of Occupational Health and Safety Yokohama Rosai Hospital, 3211, Kozukue, Kohoku, Yokohama, Kanagawa 222-0036 Japan; 2 Graduate School of Health Science, Suzuka University of Medical Science, 1001-1, Kishioka, Suzuka, Mie 510-0293 Japan; 3 Department of Radiology, Kanazawa University Hospital, 13-1, Takaramachi, Kanazawa, Ishikawa 920-8641 Japan; 4 Department of Radiology, The Jikei University Kashiwa Hospital, 163-1 Kashiwashita, Kashiwa, Chiba, 277-8567 Japan; 5 Department of Imaging Center, St. Marianna University School of Medicine Hospital, 2-16-1, Sugao, Miyame, Kawasaki, Kanagawa 216-8511 Japan; 6 Medical Quantum Science, Department of Health Sciences, Faculty of Medical Sciences, Graduate School of Medical Sciences, Kyushu University, 3-1-1, Maidashi, Higashi-ku, Fukuoka, 812-8582 Japan; 7 Department of Environmental Health, National Institute of Public Health, 2-3-6, Minami, Wako, Saitama, 351-0197 Japan; 8 Department of Radiological Technology, Teikyo University School of Medicine, University Hospital, Mizonokuchi, 3-8-3, Mizonokuchi, Takatsu-ku, Kawasaki City, Kanagawa 213-8507 Japan; 9 Department of Radiology Service, Shiga University of Medical Science Hospital, Setatsukinowa-chou, Ootsu, Shiga 520-2192 Japan; 10 Department of Radiology, Nippon Medical School Tama Nagayama Hospital, 1-7-1, Nagayama, Tama, Tokyo, 206-8512 Japan; 11 Department of Medical Technology, Tokai University of Medical Science Hospital, 143, Shimokasuya, Isehara, Kanagawa 259-1143 Japan; 12 Diagnostic Imaging, Kawasaki Municipal Tama Hospital, 1-30-37, Syukugawara, Tama-ku, Kawasaki, Kanagawa 214-8525 Japan; 13 Department of Nuclear Medicine and Tracer Kinetics, Osaka University Graduate School of Medicine, 2-2, Yamadaoka, Suita, Osaka 565-0871 Japan; 14 Department of Radiology, Kindai University Faculty of Medicine, 377-2, Ohno-Higashi, Osaka-Sayama, Osaka 589-8511 Japan

**Keywords:** Computed tomography, Shielding calculation method, Air kerma scatter factor, Dose length product, Workload

## Abstract

The goal of this study is to develop a more appropriate shielding calculation method for computed tomography (CT) in comparison with the Japanese conventional (JC) method and the National Council on Radiation Protection and Measurements (NCRP)-dose length product (DLP) method. Scattered dose distributions were measured in a CT room with 18 scanners (16 scanners in the case of the JC method) for one week during routine clinical use. The radiation doses were calculated for the same period using the JC and NCRP-DLP methods. The mean (NCRP-DLP-calculated dose)/(measured dose) ratios in each direction ranged from 1.7 ± 0.6 to 55 ± 24 (mean ± standard deviation). The NCRP-DLP method underestimated the dose at 3.4% in fewer shielding directions without the gantry and a subject, and the minimum (NCRP-DLP-calculated dose)/(measured dose) ratio was 0.6. The reduction factors were 0.036 ± 0.014 and 0.24 ± 0.061 for the gantry and couch directions, respectively. The (JC-calculated dose)/(measured dose) ratios ranged from 11 ± 8.7 to 404 ± 340. The air kerma scatter factor κ is expected to be twice as high as that calculated with the NCRP-DLP method and the reduction factors are expected to be 0.1 and 0.4 for the gantry and couch directions, respectively. We, therefore, propose a more appropriate method, the Japanese-DLP method, which resolves the issues of possible underestimation of the scattered radiation and overestimation of the reduction factors in the gantry and couch directions.

## Introduction

Prior to the installation of new X-ray equipment in medical institutions, a pre-evaluation of radiation safety must be performed to ensure that the radiation doses delivered to workers and the public are below the dose constraints imposed by international radiation safety requirements. It is important to ensure that structural radiation shielding is properly designed and installed during the original construction process, because corrections or modifications performed after the construction of the facilities are expensive [1]. It is, therefore, essential to confirm beforehand whether a given computed tomography (CT) scanner will meet the dose constraints, especially considering that recent rapid advancements in CT technologies have led to high-performance scanners with multi-row detectors, which provide increased patient throughput [2, 3].

The National Council on Radiation Protection and Measurements (NCRP) in the United States recommends the shielding calculation method described in Report No. 49 [4], published in 1976, which was revised in Report No. 147 in 2004.

For X-ray equipment other than CT scanners, Reports No. 49 and 147 both recommend a method that uses the assumed maximum workload as a parameter of the radiation source conditions. In contrast, for CT scanners, Report No. 147 recommends three CT shielding calculation methods: the NCRP-dose length product (DLP) method, the computed tomography dose index (CTDI) method, and the isodose map method. These techniques do not utilize the maximum workload.

In 2000, the British Institute of Radiology (BIR) and the Institute of Physics and Engineering in Medicine (IPEM) proposed shielding calculation methods for diagnostic X-ray techniques using general X-ray radiography and X-ray fluoroscopy, including CT, in a collaborative guideline [5]. The Japanese Ministry of Health, Labour, and Welfare recommends a method, hereafter referred to as the Japanese conventional (JC) method that is based on NCRP Report No. 49, for X-ray equipment including CT scanners [6, 7]. However, NCRP did not recommend the use of the workload, as in the JC method, in its shielding calculation method for CT.

According to Cole et al. [8] and Wallance et al. [9], the NCRP-DLP method may be the most realistic approach because the DLP is easy to acquire and is considered an appropriate indicator for calculating the radiation dose. Moreover, the NCRP states that the NCRP-DLP method is more convenient than other methods. However, the air kerma scatter factor κ for this method has not been sufficiently validated and this technique might underestimate doses [8–11]. For the shielding calculation, every evaluated point must exhibit a dose below the dose constraints. It has been confirmed that one of the issues of the NCRP-DLP method is the potential underestimation of the scattered radiation in certain situations. Furthermore, the fan angle of the beam and the beam width, which possibly affect the scattered radiation dose during scanning, have changed with technological developments; this might also lead to errors in the estimated doses, as stated in NCRP Report No. 147 as a cautionary note regarding the rapidly changing developments in CT imaging.

The goal of this study is to develop a more appropriate shielding calculation method for CT by comparatively evaluating the JC and NCRP-DLP methods based on current clinical settings.

## Materials and methods

### CT scanners

Considering the current market share in Japan [12], 18 CT scanners (seven from Toshiba Medical Systems Corporation, three from Hitachi Ltd., four from General Electric Healthcare, and four from Siemens Japan K.K.) were examined in this study and the models from each manufacturer were Aquilion CX, Aquilion 64, Prime 80, and One 320 (Toshiba Medical Systems Corporation, Tochigi, Japan); Scenaria and Eclos (Hitachi Ltd., Tokyo, Japan); VCT and Lightspeed VCT (General Electric Healthcare, Tokyo, Japan); and Definition Flash, Definition AS + , Somatom Definition Edge, and Somatom Definition Flash (Siemens Japan K.K., Tokyo, Japan); respectively.

### Measurements

For one week in December 2013, 288 optically stimulated luminescence dosimeters (OSLDs) [13] (Nagase Landauer Co., Ltd., Ibaraki, Japan) were attached to the walls of clinical CT application rooms at a height of 1 m to measure the scattered doses.

These dosimeters, often used for occupational exposure measurements and ambient dose measurements, comprise three filters such as plastic, aluminum, and copper and also have an open window. They can evaluate ambient doses using the absorption ratios of four elements and exhibit good accuracy for measurements of the energy dependency and linearity in a diagnostic field, with errors below 10%. No fading compensation was required, because no fading was observed at room temperature during the one-week period.

We used the Quixel badge service, an ambient dose equivalent measurement service using the OSLD, provided by Nagase Landauer Co., Ltd. [14]. Quixel badges were placed on a wall and the data from the read-out of the OSLDs were converted to the ambient dose equivalent for free-air exposure conditions, H*(10), according to the original standard defined by the Japanese Industrial Standards. Although the OSLDs were placed on each wall of the CT room, the doses were measured and interpreted as the ambient dose equivalent, H*(10), with a backscatter dose.

**Fig. 1:**
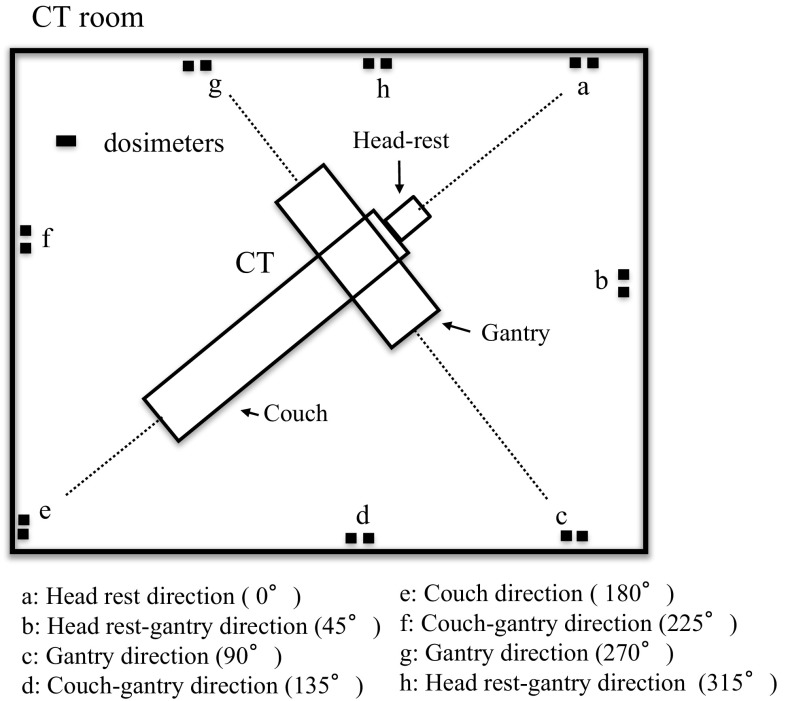
Schematic diagram of the dosimeter arrangement

**Fig. 2:**
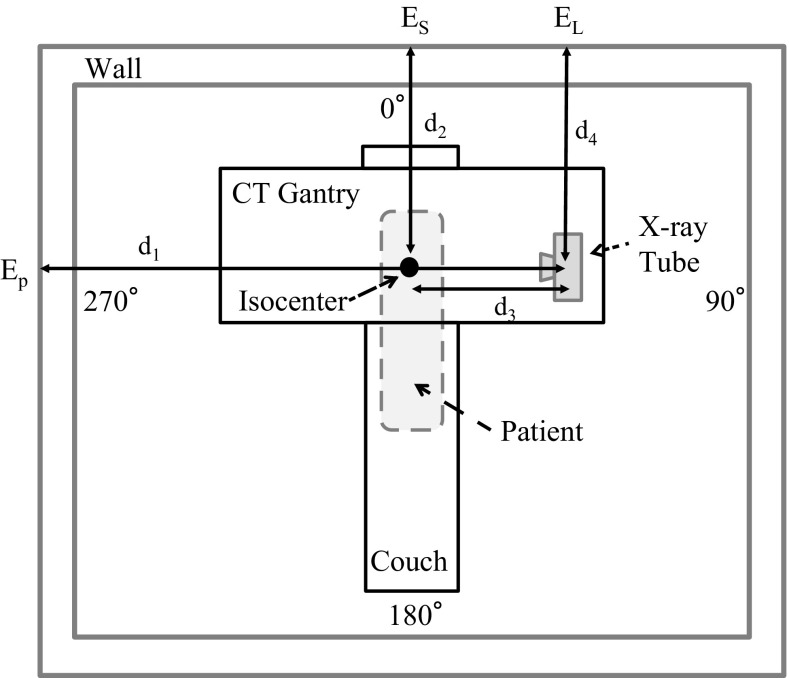
Schematic diagram of the JC method for CT shielding calculation

A schematic view of the dosimeter arrangement in the CT room is shown in Fig. 1. A pair of OSLDs was placed in each of the investigated directions: direction “a” is the head rest direction (0°), direction “e” is the couch direction (180° as defined in this article), directions “c” and “g” are gantry directions (90°, 270°), directions “b” and “h” are head rest-gantry directions (45°, 315°), and directions “d” and “f” are couch-gantry directions (135°, 225°). An additional dosimeter (OSLD) that was less susceptible to the leakage dose was placed outside the CT room to measure the background. It should be noted that for clinical reasons, the isocenter was not located at the exact center of the gantry to the couch direction (defined as 180° direction in this study, Fig. 2). When measuring scattered radiation, the effect of the gantry must be considered. Because the dosimeters at 45° and 315° were susceptible to the shielding effect of the gantry, these dosimeters were shifted slightly toward the 0° direction.

### Calculation

The scattered dose was evaluated with the NCRP-DLP and JC methods for the same period as the actual measurements.

#### NCRP-DLP method

The NCRP-DLP method utilizes Eqs. (1) and (2), which express the effective dose for head (*K*_sec_(head)) and body (*K*_sec_(body)) examinations, respectively, and then summed. The air kerma scatter factors, *k*_head_ (9 × 10^−5^ cm) and *k*_body_ (3 × 10^−4^ cm), and a constant of 1.2 were used, as recommended by the NCRP. *k*_head_ and *k*_body_ show the percentage of the amount of scattered radiation at a distance 1 m from the scattering body; the proportion as given in per unit DLP in the NCRP-DLP method utilizes Eqs. (1) and (2), respectively. There is a need to insert a distance factor of the equation to calculate the scattering radiation dose at any distance. Therefore, the distance d (cm) was added and defined in Eqs. (1) and (2). In addition, the unit of the air kerma scatter factor was changed from (cm^−1^) to (cm).

In the United States and the United Kingdom, the dose criteria are defined using the air kerma (Gy) and not by the effective dose. However, in Japan, they are defined using the effective dose (Sv), since the assessed dose should be compared with dose constrains indicated as effective doses. Consequently, the air kerma was converted to the effective dose for comparison with the dose criteria; this was also applied to the JC method using a conversion factor (*E/*Ka) of 1.433 as the maximum value considering the range of radiation energy in an X-ray room (Health Policy Bureau, MHLW Notification No. 188). The NCRP recommends multiplying the DLP by 1.4 if the ratio of the number of contrast examinations to that of non-contrast examinations is unknown. However, this was not the case in the present study because the exact number of contrast and non-contrast examinations was determined from the exposure reports provided by the hospitals.

In the NCRP-DLP method, the DLP (mGy·cm) displayed on the scanner screen was used. We required the uncertainties of CTDI_vol_ to be below 20%, according to the Japanese Industrial Standards, which are based on the International Electrotechnical Commission (IEC) 60601-2-44 ed3.0 [15], and the uncertainties of the displayed CTDI_vol_ should be below 20%. The calculated DLPs were based on actual CTDIs, considering automatic exposure control (AEC). It was thought that DLP is the most reliable indicator for the radiation dose exposed from a CT scanner, since it takes into account tube voltage, workload, and the effect of the beam width for each examination. *K*_sec_(head) and *K*_sec_(body) were calculated separately and then summed.

1 $$K_{\sec } \left( {\text{head}} \right) \, = \, k_{\text{head}} \times {\text{ DLP }} \times \, E/{\text{Ka }} \times \, \left( {1/d} \right)^{2}$$

2 $$K_{ \sec } \left( {\text{body}} \right) \, = \, 1.2 \, \times \, k_{\text{body}} \times {\text{ DLP }} \times \, E/{\text{Ka }} \times \, \left( {1/d} \right)^{2}$$

#### JC method

The JC method uses Eq. (3) to calculate the primary beam, Eq. (4) for the scattered radiation, and Eq. (5) for the leakage dose from the X-ray tube. The Japanese Ministry of Health, Labour, and Welfare recommends a shielding calculation method for X-ray equipment (general X-ray radiography, X-ray fluoroscopy, etc.), including CT scanners, that does not consider the specific characteristics of the CT scanner (NCRP Report No. 49). A schematic diagram of the shielding calculation by the JC method is shown in Fig. 2. The requirements for the shielding of each wall, ceiling, and floor must be evaluated at four X-ray tube positions (up: 0°, right: 90°, down: 180°, left: 270° in Fig. 2), typically without considering the beam time at each position. The primary beam, scattered radiation, and leakage dose from the X-ray tube are calculated at each X-ray tube position and each evaluation direction. Finally, the results are summed. In the present study, OSLDs were placed on each wall of the CT room. The shielding effects of the walls, ceiling, and floor were not evaluated. The thickness of the gantry of the CT scanner depended on the scanner; typically, it was considered equivalent to 2.5-mm-thick lead. The use factor in each direction was assumed to be 1.0. Furthermore, a conversion factor (*E*/Ka) of 1.433 was employed to obtain the effective dose, as in the DLP method.

The effective dose includes the primary beam (*E*_p_, mSv), secondary radiation (*E*_s_, mSv), and leakage from the tube housing (*E*_L_, mSv). The individual parameters are shown in Table 1.

**Table 1:** Parameters and description of the JC method^a^

Parameter	Description (unit)
*E*_p_	Effective dose due to the primary beam (mSv)
*E*_s_	Effective dose due to secondary radiations (mSv)
*E*_L_	Effective dose due to leakage from the tube housing (mSv)
*X*	Air Kerma of rate per 1 mA standardized at 1 m from the focus of X-ray tube (mGym^2^/mAs)
*Dt*	Air kerma transmission factor on barrier of thickness *t* (cm) except for filtered X-ray by primary barrier
*W*	Workload (mAs, in case of *E*_p_ mAs)
*E*/Ka	Converting factor to effective dose from air kerma (Sv/Gy)
*U*	Use factor
*T*	Occupancy factor
*d*_*1*_	Distance from the focus of X-ray tube to the point of interest for evaluation of *E*_p_ (m)
*d*_*2*_	Distance from the patient to the point of interest for evaluation of *E*_s_ (m)
*d*_*3*_	Distance from the focus of X-ray tube to the patient (m)
*d*_*4*_	Distance from the focus of X-ray tube to the point of interest for evaluation of *E*_L_ (m)
*a*	Scaled scatter fraction (scattered radiation ratio to *X*, assuming that *d*_3_ is 1 m and *F* is 400 cm^2^)
*F*	Exposure field (cm^2^)
*X*_*L*_	Leakage radiation dose rate (air kerma) from a tube housing standardized at 1 m from the housing (equal to 1 mGy/h according to the Ordinance for Enforcement of the Medical Care Act (mGym^2^/h)
*tw*	Number of hours of beam on-time (h)

^a^JC method: Japanese conventional method

3 $$E_{\text{P}} = \frac{{X \times Dt \times W \times (E/{\text{Ka}}) \times U \times T}}{{d_{1}^{2} }}$$

4 $$E_{\text{S}} = \frac{{X \times Dt \times W \times (E/{\text{Ka}}) \times U \times T}}{{d_{2}^{2} \times d_{3}^{2} }} \times \frac{a \times F}{400}$$

5 $$E_{\text{L}} = \frac{{X_{\text{L}} \times {\text{tw}} \times (E/{\text{Ka}}) \times U \times T}}{{d_{4}^{2} }} \times \left( {\frac{1}{2}} \right)^{{(\frac{t}{t1/2})}} .$$

#### DLP and workload

The IEC requires that the DLP is displayed on the console screen of a CT scanner (60601-2-44 ed3.0) [15]. However, the definition of the displayed workload (mAs) varied for the 18 CT scanners employed in this study. Two CT scanners (CT-1 and CT-2) provided the maximum workload by assuming a constant tube current at the maximum tube current during the scan, whereas others provided the actual workload, which was calculated from the archive log of the actual tube current reflecting the AEC. In the case of two CT scanners for which the actual workload could not be obtained, we used the following method to calculate the actual workload. This method was applicable for two CT scanners that provided information of the tube current for each image during a scan. For example, in the case of 5-mm slices for a 30-cm scan range, 150 images would be obtained. In this case, we recorded each tube current for each image (*n* = 150) and calculated the arithmetic mean tube current for this examination. Then, the actual workload for this examination was calculated by multiplying the mean tube current by the exposure time for this examination. However, this method was not pragmatic since it required calculating each mean workload for all examinations (approximately 1000 examinations for two CT scanners) during our study period. Therefore, we excluded the results of the two CT scanners that displayed the maximum workload.

### Statistical analysis

The statistical analysis was performed using the application Ekuseru-Toukei 2012 (Social Survey Research Information Co., Ltd., Tokyo, Japan), an add-in of Microsoft Office Excel 2013 and R version 2.14.1 [16]. The statistical difference was examined by a two-sample Student’s *t* test and the pairwise association was examined by Pearson’s correlation coefficient test (*r*). Differences with *p* < 0.05 were considered significant.

## Results

### Dose measurements

The basic information on the scanners and the DLPs and workloads that were used for the calculation is shown in Table 2. The converted dose at 1 m from the isocenter and the distance from the isocenter to the dosimeters are shown in Table 3. The measured dose (net dose considering the background level) ranged from below 0.01 (not detected, ND) to 25.15 mSv. Two doses were ND (2/144, 1.4%). The dose at 1 m from the isocenter ranged from ND to 170.67 mSv and the distance from the isocenter to the measurement direction was 158–494 cm. For scanner CT-4 (each scanner is described in detail in Table 2), the converted value at 1 m was not calculated at 45° and 225° because the doses at these directions were ND.

**Table 2:** Characteristics of examined CT scanners and comparison of DLPs and workloads (mAs)

Scanner	Manufacturer and model	Number of detector rows	Maximum beam width (cm)	Region	Total DLP^a^/week	DLP^a^/week/region	Total workload/week	Workload/week/region	Number of scans	Head examinations ratio as DLP basis^b^ (%)
CT-1	Toshiba	64	3.2	Head	429,619	147,415	–	–	146	34.3
	Aquilion CX			Body		282,204		–	290	
CT-2	Toshiba	64	3.2	Head	500,739	171,884	–	–	182	34.3
	Aquilion64			Body		328,855		–	344	
CT-3	Toshiba	64	3.2	Head	120,587	31,780	309,468	84,194	50	26.4
	Aquilion64			Body		88,807		225,274	153	
CT-4	Toshiba	80	4	Head	56,078	45,850	105,810	76,479	60	81.8
	Prime 80			Body		10,228		29,331	46	
CT-5	Toshiba	64	3.2	Head	240,577	64,314	678,783	202,591	79	26.7
	Aquilion 64			Body		176,263		476,192	249	
CT-6	Hitachi	64	4	Head	171,881	64,478	432,662	181,766	108	37.5
	Scenaria			Body		107,404		250,897	99	
CT-7	GE	64	4	Head	349,317	27,276	797,230	148,788	32	7.8
	VCT			Body		322,042		648,442	320	
CT-8	Hitachi	64	4	Head	263,440	86,603	857,834	354,165	95	32.9
	Scenaria			Body		176,837		503,669	237	
CT-9	Hitachi	16	2	Head	15,967	5369	98,827	39,907	8	33.6
	Eclos			Body		10,597		58,920	18	
CT-10	GE	64	4	Head	222,722	39,567	716,886	113,500	33	17.8
	VCT			Body		183,156		603,387	223	
CT-11	Siemens	128	3.84	Head	368,432	27,624	1,592,397	38,484	15	7.5
	Definition Flash			Body		340,808		1,553,913	464	
CT-12	GE	64	4	Head	442,330	14,316	1,472,663	49,664	17	3.2
	VCT			Body		428,014		1,422,999	480	
CT-13	Toshiba	320	16	Head	262,459	51,570	741,219	87,495	46	19.6
	One320			Body		210,889		624,837	188	
CT-14	Toshiba	320	16	Head	133,360	42,147	287,042	49,348	65	31.6
	One320			Body		91,214		237,694	154	
CT-15	Siemens	64	3.84	Head	239,687	90,459	997,378	341,063	112	37.7
	Definition AS+			Body		149,228		656,315	371	
CT-16	Siemens	64	3.84	Head	225,482	37,670	894,669	79,236	63	16.7
	Somatom Definition Edge			Body		187,811		815,433	365	
CT-17	Siemens	128	3.84	Head	162,523	26,020	692,390	65,053	41	16.0
	Somatom Definition Flash			Body		136,504		627,337	315	
CT-18	GE	64	4	Head	166,343	52,435	386,864	120,144	48	31.5
	Lightspeed VCT			Body		113,908		266,720	172	
								Mean		27.6
								Standard deviation		17.4

^a^Units of DLP is Gy cm

^b^Head examinations ratio as DLP basis was calculated as the ratio of head examination/head and body examinations

^c^It could not obtain the actual workload

**Table 3:** Converted doses and distances in the CT room

Item	Point (°)	CT-1	CT-2	CT-3	CT-4	CT-5	CT-6	CT-7	CT-8	CT-9	CT-10	CT-11	CT-12	CT-13	CT-14	CT-15	CT-16	CT-17	CT-18
Converted dose at 1 m^a^ (mSv)	0	46.74	53.80	16.08	8.31	24.09	27.09	35.06	37.23	2.23	32.83	48.50	47.93	29.94	12.73	33.59	32.02	24.13	26.79
	45	82.42	106.77	12.78	–^c^	18.88	42.24	14.71	34.81	0.88	27.64	65.12	43.98	67.25	10.14	29.30	32.27	22.06	16.94
	90	3.49	4.81	1.82	0.26	1.74	1.85	1.70	2.36	0.26	2.74	2.51	5.59	1.46	0.88	1.13	2.24	0.79	1.39
	135	97.47	166.77	48.55	11.09	48.67	37.58	71.41	52.96	5.54	66.44	85.30	170.67	55.12	26.83	18.87	44.39	50.44	45.19
	180	21.29	23.91	8.66	2.14	15.29	6.75	18.10	18.89	1.44	21.92	35.65	30.64	12.62	6.42	14.91	15.26	13.18	16.05
	225	95.22	91.05	26.49	–^c^	54.68	31.29	44.11	92.88	5.08	87.01	112.66	147.90	56.56	26.73	47.59	47.72	48.76	30.26
	270	3.56	5.90	1.53	0.44	2.02	1.25	1.22	4.54	0.24	1.72	6.06	4.64	2.89	0.82	3.92	3.48	2.51	1.46
	315	94.23	107.49	5.58	5.90	16.61	22.29	7.71	67.27	0.55	19.39	66.86	52.57	62.37	18.00	36.55	24.66	28.10	18.81
Distance^b^ (cm)	0	227	217	190	235	209	242	210	249	256	238	241	229	230	225	230	210	240	230
	45	225	238	300	235	200	200	258	197	215	280	220	296	270	175	194	300	336	185
	90	225	214	275	360	198	297	175	200	158	203	302	221	200	160	213	248	296	210
	135	383	412	383	355	296	360	270	180	233	266	383	276	315	225	193	360	424	255
	180	435	417	494	391	473	362	390	366	380	334	417	439	390	360	390	420	416	420
	225	277	239	233	329	251	293	290	340	338	186	371	357	400	470	403	458	456	360
	270	300	300	250	214	180	233	215	348	185	180	267	296	255	185	240	330	320	210
	315	198	210	300	209	222	198	180	255	280	190	260	292	270	275	223	300	344	190

^a^Dose was converted to 1 m from the isocenter

^b^Distance to the measurement position from the isocenter

^c^Not detected

### Comparison of measured and calculated doses

The calculated dose was obtained using the NCRP-DLP and JC methods. The ratios of the calculated doses to the measured (*M*) dose (NCRP-DLP/M ratio and JC/M ratio) are shown in Table 4. All the data are expressed as mean ± standard deviation (SD) and *N* is the number of CT scanners used in each group. The NCRP-DLP method delivered mean ratios for each direction ranging from 1.7 ± 0.6 (135°) to 55 ± 24 (90°) and none of the mean values was below 1. However, three of the 142 examined directions had an NCRP-DLP/M ratio below 1; that is, 3.4% (3/88) of examined directions in the directions from the subject and the gantry (i.e., 0°, 45°, 135°, 225°, and 315°) that had less shielding and 2.1% (3/142) of examined directions in all directions were underestimated. The minimum NCRP-DLP/M ratio, i.e., the most significant underestimation, was 0.6. On the other hand, the JC method resulted in ratios ranging from 11 ± 8.7 (135°) to 404 ± 340 (90°) and none of these mean values was below 1. All individual JC/M ratios exceeded 1 and ranged from 3.5 to 1409. The dose obtained using the JC method was 5.5–7.4 (mean 6.4) times higher than that determined using the NCRP-DLP method in all directions. In terms of directional dependency, the NCRP-DLP/M ratio ranged from 1.7 to 4.6 in the 0°, 45°, 135°, 225°, and 315° directions, probably owing to the lower shielding effect from the gantry or subjects, while the JC/M ratio ranged from 11 to 26. The doses obtained with the NCRP-DLP method were closer to the measured values and smaller than those obtained with the JC method.

**Table 4:** Ratio of the calculated dose to the measured dose

Point (°)	NCRP-DLP method^a^/measured dose		J C method^b^/measured dose		J C method^b^/NCRP-DLP method^a^
	Mean	SD	Mean	SD	
0	3.2	0.8	21	16	6.5
45	4.3	4.1	25	15	5.8
90	55	24	404	340	7.4
135	1.7	0.6	11	8.7	6.9
180	6.5	2.0	43	37	6.6
225	1.9	0.6	12	8.3	6.4
270	42	23	270	195	6.4
315	4.6	4.3	26	16	5.5
All	15	21	102	150	6.4

*SD* standard deviation

^a^NCRP-DLP: National Council on Radiation Protection and Measurements, USA method utilizing Dose Length Product

^b^JC: Japanese conventional method

Each measured dose was converted to the dose at 1 m from the isocenter by considering only the distance (Table 3). For the evaluation of the shielding effect from the gantry or subjects as a function of direction, each reduction factor due to these shielding effects was defined as the dose measured in a direction divided by the highest measured dose among all directions (Table 5). Because this ratio was also affected by the scattered radiation in the CT room, we treated it as the reduction factor of the gantry. Furthermore, we evaluated the scattering angle dependency of the scatter fraction considering the self-shielding of the subject’s body parts in the couch directions. In the gantry (90°, 270°) and couch (180°) directions, the reduction factors were smaller than in the other directions; the mean reduction factors were 0.031 ± 0.009, 0.041 ± 0.017 (mean reduction factor for gantry: 0.036 ± 0.014), and 0.240 ± 0.061, respectively, which means that the shielding effect was the highest in the gantry directions. The maximum reduction factors among all scanners were 0.082 and 0.355 for the gantry and couch directions, respectively.

**Table 5:** Reduction factors^a^ in the gantry and couch directions

Direction	Point (°)	CT-1	CT-2	CT-3	CT-4	CT-5	CT-6	CT-7	CT-8	CT-9	CT-10	CT-11	CT-12	CT-13	CT-14	CT-15	CT-16	CT-17	CT-18	Mean	SD
Gantry	90	0.036	0.029	0.037	0.023	0.032	0.044	0.024	0.025	0.047	0.031	0.022	0.033	0.022	0.033	0.024	0.047	0.016	0.031	0.031	0.009
	270	0.036	0.035	0.032	0.039	0.037	0.030	0.017	0.049	0.043	0.020	0.054	0.027	0.043	0.031	0.082	0.073	0.050	0.032	0.041	0.017
Couch	180	0.218	0.143	0.178	0.193	0.280	0.160	0.253	0.203	0.261	0.252	0.316	0.180	0.188	0.239	0.313	0.320	0.261	0.355	0.240	0.061

*SD* standard deviation

^a^The reduction factors were calculated as “dose at each direction/the highest dose” converting doses at 1 m from the isocenter shown in Table 3

## Discussion

### Dose distribution in CT room and reduction factors for gantry and couch directions

The scattered dose per DLP at a distance of 1 m from the isocenter was significantly lower in the 180° direction than in the 0° direction (*p* < 0.001). This was because of the shielding effect of the subject’s body, which is not present during cylindrical acrylic phantom measurements.

The effect of the shielding in the shielding calculation method must be properly evaluated. The results shown in Table 5 indicate a reduction factor in the gantry and couch directions. In the gantry direction, the minimum reduction factor was 0.082. Differences in the internal structure of scanners built by different manufacturers might cause changes in the shielding ratio; however, in the present study, which involved 18 scanners from four manufacturers, we can expect a reduction factor of at least 0.1 in the gantry direction. Furthermore, in the couch direction, a minimum shielding effect of 0.355 was observed. Although the ratio associated with self-shielding in the couch direction also depends on the subjects and the examined part of the body, in the present study, a dose reduction factor of at least 0.4 can be expected in the couch direction at the bed level. Thus, by introducing the reduction factor to the NCRP-DLP method, the estimated radiation dose will be closer to the true value in the gantry and couch directions. It is particularly difficult to adequately estimate the reduction factor in the couch direction without performing a multicenter study with clinical settings.

### Issues with the NCRP-DLP method

**Fig. 3:**
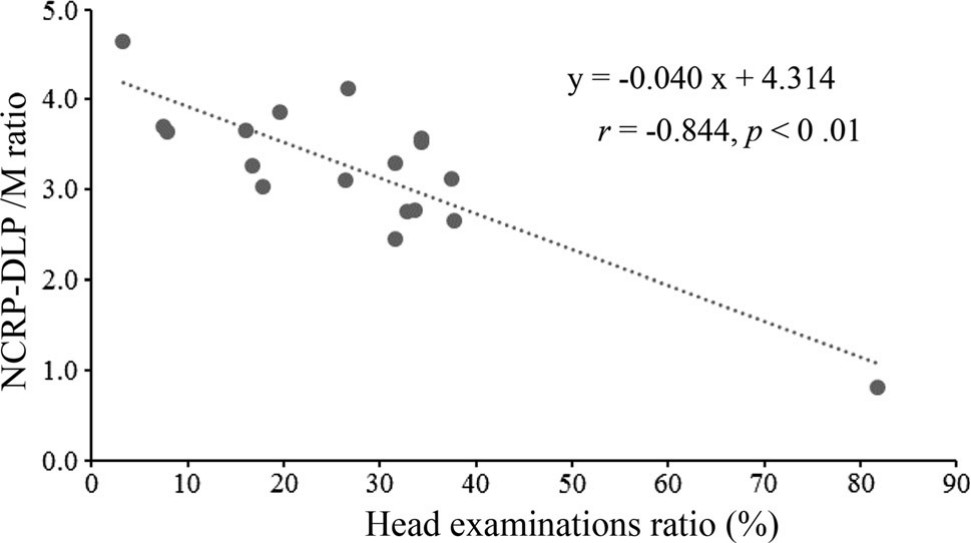
Relationship between head ratio and NCRP-DLP/M ratio in the case of the 0° direction

The doses assessed with the NCRP-DLP method were more consistent with the measured doses than those obtained with the JC method. However, 3.4% (3/88) of the measured doses were underestimated in the directions from the gantry and subject that had less shielding. The underestimated NCRP-DLP/M ratios were 0.6 and 0.8 (scanner CT-4, 135° and 0°) and 0.9 (scanner CT-6, 45°). CT scanners CT-4 and CT-6 were mostly used for head examinations (the ratio of head examinations was 82% for scanner CT-4 and 38% for scanner CT-6, with a DLP basis), while for other CT scanners, the usage ratio of head examinations was 28 ± 17% with a DLP basis, as indicated in Table 2. Furthermore, although only in the case of the 0° direction, a statistically significant correlation was not observed between the head examinations ratio and measured dose (*r* = 0.298, *p* = 0.43), but a statistically significant correlation was observed between head examinations ratio and NCRP-DLP/M (*r* = 0.844, *p* < 0.01). Moreover, the NCRP-DLP/M ratio was significantly reduced along with the head ratio (Fig. 3). In the NCRP-DLP method, the air kerma scatter factor κ was calculated from the air kerma scatter factors of the head and body, which were subsequently summed, as stated in Sect. 2.3.1 of the report that describes the NCRP-DLP method [1]. In other words, in the NCRP-DLP method, the air kerma scatter factor κ for the head is considered relatively low compared to that for the body.

In addition, the results of this study were consistent with the value of 0.7 as the NCRP-DLP/M ratio reported by Cole et al. (clinical research; one scanner per manufacturer, three scanners) [8]. Cole et al. suggested that calculation methods such as NCRP-DLP perform better with head examinations than with body examinations. Similarly to the results of that study, which reported a smallest NCRP-DLP/M ratio of 0.7 for mostly head examinations, the smallest calculation ratio in our study was observed for the CT scanner that was used mostly for the head examinations (as DLP basis: 82%), while for other CT scanners, the usage ratio was 28 ± 17% as DLP basis as indicated in Table 2.

Based on these results including the present results and those of [8], particular attention is recommended for calculations performed for head examinations.

As mentioned in the introduction, several papers have indicated that the NCRP-DLP method can underestimate the air kerma scatter factor κ and the associated calculation of the air kerma scatter factor κ has not been sufficiently validated.

These results are consistent with those of these previous studies [8–11]. In shielding calculations, it is very important to ensure that the dose at each evaluated point is not underestimated.

Considering these issues, the possibility of underestimation by the NCRP-DLP method was not excluded and the authors believe that the air kerma scatter factor κ should, conservatively, be set on the side of safety to avoid underestimation.

It must be noted that, in this study, the air kerma scatter factor κ was not studied independently for the head and body, because the corresponding scattered doses were not measured separately. Regarding the probability of these underestimations, the measured doses of the three underestimated directions (0.6, 0.8, and 0.9) were 0.88, 1.51, and 10.56 mSv, respectively, and the detection limit of measurements with an OSLD dosimeter is 0.01 mSv. Therefore, the measured doses were sufficiently high to be detected.

### Issues with the JC method

In the JC method, the mean ratio in each direction was 5.5–7.4 (mean 6.4) times higher than that obtained with the NCRP-DLP method. The scattered dose was higher for a beam width of 16 cm than for a beam width of 4 cm in all directions except 225° (*p* < 0.01). These results indicate that the JC method involves issues with the beam width factor, as described in Eq. (4). In addition, the JC method overestimated the dose by 11–404 times in all directions. Overestimations lead to a waste of shielding resources. Therefore, realistic dose calculations must be established when considering the beam width factor.

### Issues concerning the workload, CTDI, and DLP

Depending on the part of the body being examined, the AEC is often used to modulate the tube current during the scan to optimize the dose; therefore, the actual workload is difficult to assess [17]. In addition, the ratio of the mean tube current to the maximum tube current varies depending on the body size of the subjects, scanners, manufacturers, and irradiation conditions, as described in “Materials and methods”.

Scanners currently in use worldwide employ either of two methods for assessing the CTDI, which are described as follows. The IEC has recommended the maximum value of CTDI_vol_ in IEC60601-2-44 ed2.1 [18]. In 2010, the IEC recommended the mean value for the tube current in IEC60613 [19]. Therefore, whether the maximum or average CTDI_vol_ is indicated on the console screen of a scanner’s display depends on when the scanner was manufactured. On the other hand, the IEC requires that the scanner console screens display the DLP on the basis of the average CTDI_vol_ during a scan (60601-2-44 ed3.0) [15]. Before this recommendation, such as IEC60601-2-44 ed2.1, the IEC did not recommend to display and record the DLP for a CT scan. Therefore, in shielding calculations, the use of the DLP that is displayed on the scanner console screen is more reliable than that calculated from CTDI_vol_.

### Proposal of new Japanese-DLP method

Because the air kerma scatter factors can be underestimated by the NCRP-DLP method, we propose a head and body air kerma scatter factor that is twice as high as that used in the NCRP-DLP method; additionally, the dose reduction factors should be 0.1 and 0.4 for the gantry and couch directions, respectively, considering numerical rounding for safety reasons.

The ratio of the doses calculated with the Japanese-DLP method to the measured dose is shown in Table 6. This modified method that is based on the NCRP-DLP method is hereafter referred to as the Japanese-DLP method. The mean Japanese-DLP dose/measured dose (Japanese-DLP/M) ratio in each direction ranged from 3.3 (135°) to 11 (90°) (mean 7.0). Moreover, we confirmed that the minimum value was 1.2 and the values at all directions were above 1.

**Table 6:** Ratio of the doses calculated with the Japanese-DLP method and the NCRP-DLP method to the measured dose

Point (°)	Japanese-DLP method^a^/measured dose		NCRP-DLP^b^/measured dose		NCRP-DLP method^b^/Japanese-DLP method^a^
	Mean	SD	Mean	SD	
0	6.4	1.6	3.2	0.8	0.5
45	8.5	8.2	4.3	4.1	0.5
90	11.0	4.8	55	24	5.0
135	3.3	1.1	1.7	0.6	0.5
180	5.2	1.6	6.5	2.0	1.3
225	3.8	1.2	1.9	0.6	0.5
270	8.4	4.5	42	23	5.0
315	9.3	8.6	4.6	4.3	0.5
All	7.0	2.8	15	21	2.1

*SD* standard deviation

^a^Japanese-DLP: Japanese-Dose Length Product method

^b^NCRP-DLP: National Council on Radiation Protection and Measurements, USA method utilizing Dose Length Product

### Comparison of shielding calculation methods

Compared to the JC method, the NCRP-DLP method has the following advantages: (1) the calculated values are more consistent with the measured values, (2) the main parameter (DLP) used can be more clearly defined, (3) it is less susceptible to fluctuations due to AEC, and (4) it is less sensitive to the number of detector rows (beam width), as mentioned in the NCRP Report No. 147 (“Attempting to utilize a workload expressed in mA min week^−1^ is not recommended”.). The results of the present study indicate that the NCRP-DLP method has fewer problems than the JC method and is, therefore, more reliable.

A comparison of derived required thickness for shielding between each shielding calculation method and measurements is shown in Table 7.

**Table 7:** Comparison of required thickness for shielding of the scanner “CT-13^a^” calculated by each method as a typical example

Scanner	Point (°)	Evaluated dose (mSv/3 M)^b^				Ratio of JC^d^ to Measured	Required shielding ratio^f^				Required thickness for shielding (Pb, mm)^g^				JC-Measured^h^ (Pb, mm)
		NCRP-DLP^c^	J C^d^	Japanese-DLP^e^	Measured		NCRP-DLP^c^	J C^d^	Japanese-DLP^e^	Measured	NCRP-DLP^c^	J C^d^	Japanese- DLP^e^	Measured	
CT-13	0	2.8E+02	5.0E+03	5.7E+02	7.4E+01	6.8E+01	4.6E−03	2.6E−04	2.3E−03	1.8E−02	1.3	2.5	1.6	0.9	1.6
	45	2.1E+02	3.6E+03	4.1E+02	1.2E+02	3.0E+01	6.3E−03	3.6E−04	3.2E−03	1.1E−02	1.2	2.4	1.5	1.0	1.4
	90	3.8E+02	6.7E+03	7.5E+01	4.7E+00	1.4E+03	3.5E−03	1.9E−04	1.7E−02	2.7E−01	1.5	2.7	0.9	0.2	2.5
	135	1.5E+02	2.7E+03	3.0E+02	7.2E+01	3.7E+01	8.6E−03	4.9E−04	4.3E−03	1.8E−02	1.1	2.3	1.4	0.9	1.4
	180	9.9E+01	1.7E+03	7.9E+01	1.1E+01	1.6E+02	1.3E−02	7.5E−04	1.6E−02	1.2E−01	1.0	2.1	0.9	0.4	1.7
	225	9.4E+01	1.7E+03	1.9E+02	4.6E+01	3.6E+01	1.4E−02	7.8E−04	6.9E−03	2.8E−02	0.9	2.1	1.2	0.7	1.4
	270	2.3E+02	4.1E+03	4.6E+01	5.8E+00	7.1E+02	5.6E−03	3.2E−04	2.8E−02	2.2E−01	1.3	2.4	0.7	0.2	2.2
	315	2.1E+02	3.6E+03	4.1E+02	1.1E+02	3.3E+01	6.3E−03	3.6E−04	3.2E−03	1.2E−02	1.2	2.4	1.5	1.0	1.4
Mean							7.7E−03	4.4E−04	1.0E−02	8.8E−02	1.2	2.4	1.2	0.7	1.7
SD							3.9E−03	2.2E−04	9.4E−03	1.1E−01	0.2	0.2	0.3	0.3	0.4

*SD* standard deviation

^a^The detailed of the scanner “CT-13” is described in Table 2

^b^Dose was converted from a week dose to three months dose at evaluated points

^c^NCRP-DLP: The method using Dose Length Product described in National Council on Radiation Protection and Measurements-Dose Length Product

^d^JC: Japanese conventional calculation method

^e^Japanese-DLP: Japanese-Dose Length Product method

^f^Required Shielding ratio: Minimum transmission for shielding at the boundary of controlled area [below 1.3 (mSv/3 M)]

^g^Required thickness for shielding: Minimum thickness of shielding to achieve the standard for the boundary of controlled area (below 1.3 (mSv/3 M))

^h^JC-Measured: JC-measured was calculated as “required thickness for shielding by JC”−“required thickness for shielding by measured”

The results indicate that the JC method mostly overestimated the shielding thickness among these calculation methods. The JC method calculated two times thicker shielding than the NCRP-DLP and Japanese-DLP methods.

On the other hand, though the mean shielding thickness required is the same in the NCRP-DLP and Japanese-DLP methods, the Japanese-DLP method has the advantage of not underestimating at any points, as mentioned in the previous section.

In the average of the “ratio of calculated dose to measured dose” of all directions in Table 6, the Japanese-DLP method is approximately half of the NCRP-DLP method. On the other hand, NCRP-DLP method overestimates in the 90° and 270° directions, compared to the Japanese-DLP method.

Among 142 evaluation points, the ratios of the measured to calculated doses were underestimated at three points in the case of the NCRP-DLP method in our study. How should this risk be evaluated? We think that it would be dependent on the individual countries and regions, since the basic concept of the shielding calculation method would be related to local cultures. Moreover, we believe that every evaluated point must exhibit a dose that is below the dose constraints for the shielding calculation in Japan. Furthermore, in Japan, because strict defense of the dose constraints is required by the public, we had proposed to double the air kerma scatter factor. Similarly, the JC method overestimated leaked radiation. In other words, the conceptual bases of the Japanese-DLP and NCRP-DLP methods might be different.

In addition, we had proposed the reduction factor by conservatively rounding the observed minimum reduction factors of the gantry and subjects. Moreover, it may be possible to set the reduction factors as the mean + 2SD of measured results, depending on the situation of the countries and regions.

Table 8 shows the results of the comparison between the shielding calculation methods. Considering the widespread use of AEC during CT scans, studies are currently performed on shielding calculation methods using cylindrical acrylic phantoms and the Rando phantom. In particular, CT dose optimization techniques are under development and the evaluation of the scattered radiation doses is currently insufficient. In the present multicenter study, we increased the sample size to sufficiently evaluate the NCRP-DLP method and the reduction factors in the gantry and self-shielding directions. Therefore, shielding calculation studies should be conducted in a multicenter study framework and performed conservatively to ensure radiation safety. We should note that previously performed conventional shielding calculation studies might have potential limitations owing to the limited number of assessed scanners and facilities.

**Table 8:** Comparison of shielding calculation methods

Methods	NCRP-DLP^a^ [8]	NCRP-DLP^a^ [9]	Japanese-DLP^b^ (present study)	Japanese conventional^c^ (present study)
Number of manufacturers	3	4	4	4
Number of facilities	3	Do not show	12	11
Number of scanners	3	4	18	16
Research type	Clinical	Rando phantom	Clinical	Clinical
Minimum calculated/measured ratio	0.7	Do not show	0.6	3.5
Percentage of underestimation except gantry and couch directions	22.2	Do not show	3.4	0.0
Reduction ratios of gantry	No	Yes	Yes	No
Reduction ratios of self-shield (couch direction)	No	No	Yes	No
Major parameter	DLP	DLP	DLP	mAs

^a^NCRP-DLP: National Council on Radiation Protection and Measurements, USA method utilizing Dose Length Product

^b^Japanese-DLP: Japanese-Dose Length Product method

^c^Japanese conventional: Japanese conventional method

### Limitations

This study provides new findings on the scattered radiation at multi-slice scanners with AEC; however, the associated shielding calculations involve several variables, such as the wall transmission factor, which are not discussed in this paper.

The IEC defines the acceptable variation range for the DLP as ±20% [15]. Therefore, this variance would increase the uncertainty of the NCRP-DLP evaluation. In this study, we evaluated the NCRP-DLP method conservatively to ensure radiation safety by comparing measured doses, because the measured doses were assessed without adjusting the backscattering from the wall and they overestimated the required thickness. Further studies on the backscatter factor and the variation in DLP will improve the accuracy of radiation measurements and reduce the uncertainty in radiation safety assessments.

## Conclusion

We propose a new shielding calculation method for CT, named the Japanese-DLP method, which resolves the issues of possible underestimation of the scattered radiation and overestimation of the reduction factors in the gantry and couch directions. Additionally, the proposed technique avoids the usage of workloads estimated by the CT operator that could be less reliable for automatic current modulation exposures.

The Japanese-DLP method is more appropriate than the NCRP-DLP and JC methods, especially for contemporary CT scanners.
